# Advances in Hepatitis E Virus

**DOI:** 10.3390/pathogens12080987

**Published:** 2023-07-28

**Authors:** Nora A. Fierro

**Affiliations:** Departamento de Inmunología, Instituto de Investigaciones Biomédicas, Universidad Nacional Autónoma de México, Ciudad Universitaria, Mexico City 04510, Mexico; noraalma@iibiomedicas.unam.mx

In the late 1970s, 52,000 pregnant women died in Kashmir, India. This tragic and disconcerting event, especially given the precarious economic and social reality of the affected community, was the result of a large-scale, common-source, water-borne epidemic of hepatitis. The limited infrastructure and resources in the region challenged researchers investigating the epidemic. Since all patients lacked markers of acute hepatitis A virus and hepatitis B virus, the existence of a new disease called non-A non-B hepatitis (NANBH) was announced [1].

The investigators in Kashmir, headed by Dr. Mohammed Khuroo, collaborated with Dr. Robert Purcell at NIH to identify the new virus causing the outbreak. Time passed, and in Russia, Dr. Balayan inoculated himself with samples recovered from a hepatitis outbreak in a regiment of Russian soldiers in Afghanistan. This led to the first identification of virus-like particles in 1983 and represented a decisive step toward the characterization of this etiological agent of hepatitis [1,2]. 

Viral particles in stool samples recovered during outbreaks in Mexico, together with isolates from Pakistan and Burma, allowed the description and classification of the molecular characteristics of the new hepatitis E virus (HEV) in early 1990. Later, the identification of swine HEV led to the characterization of viral diversity and underscored the importance of zoonosis in the transmission of this virus. Very importantly, the discovery of HEV as the causative agent of an intriguing chronic liver disease in transplant patients in 2010 highlighted its ability to cause chronic infection in immunosuppression settings [1,2,3]. More recently, the notion that hepatitis E might be considered a systemic disease rather than solely a liver disease was supported by the adverse effects on diverse tissues attributed to the infection, and importantly, by the ability of HEV to infect multiple cell types, including peripheral blood mononuclear cells, enterocytes, endometrial cells, and placental cells [4,5,6,7].

Currently, it is accepted that HEV infection, initially considered a poverty predictor, is also a major problem in high-income regions because of its zoonotic transmission. Indeed, HEV circulates practically worldwide, due, in part, to the diversity in its transmission routes, which in turn are related to specific viral variants. This RNA virus belongs to the *Hepeviridae* family and the *Paslahepevirus* genus. The *Paslahepevirus balayani* species includes genotypes (gts) HEV-gt1 and HEV-gt2, which are restricted to infecting humans, while HEV-gt3 and HEVgt-4 infect both humans and diverse animal species, with pigs being the main source from which zoonosis has been studied. HEV-gt7 infects camels and can also infect humans. All genotypes can be transmitted via the fecal–oral route or through contaminated blood derivatives and through the vertical route from mother to fetus. HEV-gt1 and HEV-gt2 are responsible for large, waterborne, fecal–oral outbreaks in low-income regions; HEV-gt3 and HEV-gt4 are responsible for most of the sporadic cases of hepatitis in industrialized countries. Recently, an HEV zoonosis via rat was demonstrated; this HEV was shown to belong to the *Rocahepevirus ratti* species [8].

HEV can be present either as a naked particle or as a quasienveloped virion [9]. Both forms of the virus are infectious and allow HEV dissemination. Importantly, the quasienvelope allows for the evasion of the host immune response [10] and may facilitate the dissemination of the virus; this might be related to the distribution of the virus in different tissues and the multiple extrahepatic manifestations related to the infection. In humans, the progression of HEV infection in the liver is restricted by host immunity [11]; however, the exact mechanisms responsible for the diverse outcomes associated with the infection and the pathophysiological mechanisms behind the development of extrahepatic manifestations remain to be elucidated.

The diverse forms of HEV that can infect humans make it a pathogen of global relevance. Indeed, the spread of this virus is not dictated by wealth or poverty. However, despite the milestone in the discovery of HEV, the infection is still neglected in multiple countries. There is no approved drug for the treatment of hepatitis E, and although the benefits of a vaccine licensed in China in 2011 have been shown in recent outbreaks in Africa, it is not widely available [12].

Considering the WHO’s recommendations for eliminating infectious hepatitis by 2030, joint efforts are required to improve the diagnosis and therapy of hepatitis E with viral eradication as the goal. The characterization of immune signatures associated with disease progression, extrahepatic manifestations, the design of experimental models, and the epidemiology of HEV is necessary. This Special Issue of *Pathogens* aims to collect the most recent advancements in the study of HEV. The valuable involvement of scientists worldwide with distinct points of view regarding this underestimated pathogen is expected.
